# Characterization of CPH:SA microparticle‐based delivery of interleukin‐1 alpha for cancer immunotherapy

**DOI:** 10.1002/btm2.10465

**Published:** 2022-12-07

**Authors:** M. M. Hasibuzzaman, Rui He, Ishrat Nourin Khan, Rasna Sabharwal, Aliasger K. Salem, Andrean Llewela Simons‐Burnett

**Affiliations:** ^1^ Interdisciplinary Graduate Program in Human Toxicology University of Iowa Iowa City IA USA; ^2^ Department of Pathology University of Iowa Iowa City IA USA; ^3^ Department of Pharmaceutical Sciences and Experimental Therapeutics University of Iowa Iowa City IA USA; ^4^ Department of Internal Medicine University of Iowa Iowa City IA USA; ^5^ Department of Neuroscience & Pharmacology University of Iowa Iowa City IA USA; ^6^ Holden Comprehensive Cancer Center University of Iowa Iowa City IA USA; ^7^ Department of Oral Pathology, Radiology and Medicine University of Iowa Iowa City IA USA

**Keywords:** cancer, CPH:SA, cytokine storm, cytokines, HNSCC, hypotension, inflammation, interleukin‐1 alpha, microparticles, polyanhydride, toxicity

## Abstract

**Background:**

Interleukin‐1 alpha (IL‐1α) is a pro‐inflammatory cytokine that can activate immune effector cells and trigger anti‐tumor immune responses. However, dose‐limiting toxicities including cytokine storm and hypotension has limited its use in the clinic as a cancer therapy. We propose that polymeric microparticle (MP)‐based delivery of IL‐1α will suppress the acute pro‐inflammatory side effects by allowing for slow and controlled release of IL‐1α systemically, while simultaneously triggering an anti‐tumor immune response.

**Methods:**

Polyanhydride copolymers composed of 1,6‐bis‐(p‐carboxyphenoxy)‐hexane:sebacic 20:80 (CPH:SA 20:80) was utilized to fabricate MPs. Recombinant IL‐1α (rIL‐1α) was encapsulated into CPH:SA 20:80 MPs (IL‐1α‐MPs) and the MPs were characterized by size, charge, loading efficiency, and in‐vitro release and activity of IL‐1α. IL‐1α‐MPs were injected intraperitonially into head and neck squamous cell carcinoma (HNSCC)‐bearing C57Bl/6 mice and monitored for changes in weight, tumor growth, circulating cytokines/chemokines, hepatic and kidney enzymes, blood pressure, heart rate, and tumor‐infiltrating immune cells.

**Results:**

CPH:SA IL‐1α‐MPs demonstrated sustained release kinetics of IL‐1α (100% protein released over 8–10 days) accompanied by minimal weight loss and systemic inflammation compared to rIL‐1α‐treated mice. Blood pressure measured by radiotelemetry in conscious mice demonstrates that rIL‐1α‐induced hypotension was prevented in IL‐1α‐MP‐treated mice. Liver and kidney enzymes were within normal range for all control and cytokine‐treated mice. Both rIL‐1α and IL‐1α‐MP‐treated mice showed similar delays in tumor growth and similar increases in tumor‐infiltrating CD3+ T cells, macrophages, and dendritic cells.

**Conclusions:**

CPH:SA‐based IL‐1α‐MPs generated a slow and sustained systemic release of IL‐1α resulting in reduced weight loss, systemic inflammation, and hypotension accompanied by an adequate anti‐tumor immune response in HNSCC‐tumor bearing mice. Therefore, MPs based on CPH:SA formulations may be promising as delivery vehicles for IL‐1α to achieve safe, effective, and durable antitumor responses for HNSCC patients.

## 1 BACKGROUND

Cytokines are small cell‐signaling proteins that regulate innate and adaptive immune responses. Cytokines are highly important for tumor immunosurveillance and the use of cytokine therapy to activate the immune system of cancer patients is an important treatment modality.^1^, ^2^ Various cytokines have been tested as anti‐cancer agents in preclinical and clinical studies.^3^, ^4^, ^5^, ^6^, ^7^ These studies led to the approval of interferon‐alpha (IFNα) for hairy cell leukemia in 1986 and it is now also approved for use in follicular lymphoma, melanoma, renal cell carcinoma, cervical intraperitoneal neoplasms and AIDS‐related Kaposi's sarcoma.^8^, ^9^, ^10^, ^11^ Additionally, high‐dose interleukin‐2 (IL‐2) was approved for the treatment of metastatic renal cell carcinoma in 1992, and metastatic melanoma (MM) in 1998.^12^, ^13^, ^14^, ^15^, ^16^ Clinical use of these cytokines for the treatment of cancer was among the first lines of evidence that the immune system can be modulated to mount significant anti‐tumor immune responses.

The cytokines interleukin‐1 alpha (IL‐1α) and interleukin‐1 beta (IL‐1β) have also been investigated as anti‐cancer agents in preclinical and clinical studies.^17^ Both IL‐1α and IL‐1β bind to the IL‐1 receptor type 1 (IL‐1R1) on multiple types of cells which rapidly triggers NFkB and MAPK signaling leading to the expression of numerous pro‐inflammatory cytokines (including IL‐1β, IL‐2, IL‐6, CCL2, MCP1, TNFα and IFNγ).^18^, ^19^ IL‐1 signaling was initially proposed as a key mediator of host defense against malignancies through its role in natural killer (NK) cell activity (i.e., IFNγ production and ADCC).^20^, ^21^, ^22^, ^23^, ^24^ Additionally, IL‐1 signaling was shown to directly enhance survival of CD4+ T cells and induce secondary CD8+ T cell responses characterized by enhanced granzyme B expression and increased IFNγ production.^25^, ^26^, ^27^, ^28^, ^29^, ^30^, ^31^, ^32^, ^33^ Furthermore, DCs were shown to be activated via IL‐1R1 signaling.^34^, ^35^, ^36^, ^37^ Given that IL‐1 signaling is important for DC, NK and T‐cell activity, a broad anti‐tumor immune response can be induced by stimulating IL‐1 signaling on these immune cells in cancer patients.

Unfortunately, the use of IL‐1α or IL‐1β as anti‐cancer agents in clinical trials have not lived up to the initial excitement caused by the observed preclinical anti‐tumor immune responses. Clinical studies were conducted in the late 1980s and early 1990s in cancer patients. Recombinant IL‐1 ligands (marketed as Dainippon/Immunex [IL‐1α] and Syntex [IL‐1β]) were administered to cancer patients with the most common symptoms being flu‐like symptoms including fever, chills, rigors and nausea.^17^ However, hypotension was a problematic dose‐limiting toxicity with 3 of 5 patients experiencing hypotension at the maximum tolerated dose (MTD) of 0.1 and 0.3 μg/kg (with pressors for blood pressure support).^17^ IL‐1 ligand‐induced hypotension resulted from a significant drop in systemic vascular resistance (SVR) leading to a rise in heart rate, and cardiac output rose to compensate for the drop in blood pressure and SVR.^17^ It is reported that the cardiovascular effects of IL‐1α were similar to that of septic shock and further plans for clinical trials for cancer therapy were discontinued.^17^ Therefore, managing toxicity of cytokine therapy is critically important for progressing from bench to bedside.

Controlled release systems using polymeric nano/micro‐particles may provide an appropriate strategy to infuse low levels of IL‐1 ligands (and other cytokines) over a longer period of time to cancer patients and also protect from the deleterious side effects associated with large bolus doses. Synthetic polymeric microparticles (MPs) such as poly(lactide‐co‐glycolide) (PLGA), polyanhydrides, and poly‐(diaminosulfide)s (PNSN) are examples of drug delivery vehicles that have grown in interest in the cancer immunotherapy field. Polymeric MPs have been investigated for the delivery of checkpoint inhibitors, engineered T cells, co‐stimulatory receptor agonists and cancer vaccines.^38^, ^39^, ^40^, ^41^

Polyanhydrides are an example of surface eroding polymers that are highly preferred for drug delivery strategies because of the accurate predictability of the erosion process.^42^, ^43^, ^44^ This surface erosion process contributes to a constant release rate of the drug as well as enhanced stability of the drug in the polymer.^42^, ^43^ More importantly, polyanhydrides are the only class of surface eroding polymers that are Food and Drug Administration (FDA) approved. Polyanhydrides can also be used for cytokine therapy with a goal for reduced toxicities while retaining therapeutic activity. Polyanhydride copolymers composed of 1,6‐bis‐(p‐carboxyphenoxy)‐hexane: sebacic acid 20:80 (CPH:SA 20:80) were used in this study to encapsulate IL‐1α. Here we evaluated the safety profile of CPH:SA‐based IL‐1α‐MPs compared to free (unencapsulated) IL‐1α in experimental animals. Results showed that CPH:SA‐based IL‐1α‐MPs generated a slow and sustained systemic release of IL‐1α resulting in reduced toxicity (weight loss, cytokine storm, and hypotension) compared to free IL‐1α in tumor‐bearing mice. Therefore CPH:SA formulations may be promising as delivery vehicles for IL‐1α to achieve safe and effective antitumor responses for cancer patients.

## 2 MATERIALS AND METHODS

### 2.1 Fabrication of microparticles loaded with IL‐1α

Interleukin‐1α‐microparticles (IL‐1α‐MPs) were fabricated by a water‐in‐oil‐in‐water (w/o/w) double emulsion solvent evaporation method that has been described previously with some modifications.^40^ Briefly, 167 μg of murine rIL‐1α was dissolved in 100 μl of 1% PVA solution (internal water phase, W1). The CPH:SA 20:80 polymer (200 mg) was dissolved in 1.5 ml of dichloromethane (oil phase, O). The primary emulsion (W1/O) was made by adding W1 to O under sonication using a Qsonica sonicator equipped with an ultrasonic converter probe (CL‐18, Fisher Scientific) set on 60% amplitude for 30 s. The primary emulsion was then immediately added to 8 ml of 1% PVA solution (external water phase, W2) under the same sonication condition for 60 s. The obtained W1/O/W2 emulsion was then immediately added into 22 ml of 1% PVA solution and stirred at room temperature in a fume hood for 2 h to allow evaporation of dichloromethane. The microparticles were collected by centrifugation at 5000 × *g* for 5 min (Sorvall Legend XTR, Fisher Scientific) and washed twice with nanopure water. The obtained particles were resuspended in 2 ml of 10% sucrose solution. The particle suspensions were frozen at −80°C and then lyophilized using a FreeZone 4.5 freeze dry system (Labconco Corporation) at −53°C and 0.045 mbar pressure. The lyophilized microparticles were stored in sealed containers at −20°C. Blank microparticles were fabricated using the same process. For the second dose escalation study, CPH:SA microparticles with increased IL‐1α loading were prepared following the above procedure with some modifications, where 550 μg of IL‐1α was dissolved in 100 μl of 1% PVA solution to form W1, and the final particles were resuspended in 2 ml of 5% sucrose instead of 10% sucrose. Characterization data is shown in Table 1.

**TABLE 1:** Characteristics of IL‐1α‐loaded CPH:SA microparticles.

Microparticles	Average particle size (nm ± sd)	Polydispersity index (PDI)	Zeta potential (mV)	Loading capacity of IL‐1α (μg/mg of particles)	Encapsulation efficiency (%)
1st batch particle	1011 ± 60	0.234 ± 0.024	−18.5 ± 0.3	0.18	37.9
2nd batch particle	1011 ± 84	0.235 ± 0.027	−16.9 ± 0.4	0.70	34.1

Abbreviation: sd, standard deviation.

### 2.2 Characterization of CPH:SA IL‐1α‐MPs

The MPs were resuspended in nanopure water for characterization. Size distribution and zeta potential of the MPs were measured using a Zetasizer Nano‐ZS (Malvern) by dynamic light scattering. Particle suspension was added to a polystyrene cuvette (Sarstedt Inc.) and folded capillary cell (Malvern) to determine size and zeta potential, respectively. The shape and surface morphology of MPs were determined by scanning electron microscopy (SEM) (Hitachi High Technologies). A drop of particle suspension was transferred onto a silicon wafer mounted on a SEM stub and dried completely at room temperature. Samples were coated with gold palladium using an argon beam K550 sputter coater (Emitech Ltd.). SEM images were taken using a Hitachi S‐4800 SEM at 5 kV accelerating voltage. To measure the loading of IL‐1α ‐MPs, MPs were degraded with 0.5 N NaOH. Solutions were centrifuged at 5000 × *g* for 5 min (Eppendorf Centrifuge 5424, Eppendorf AG), and the supernatants were neutralized by HCl to approximately pH 7.0. The blank MPs were treated with the same procedure as the controls. The concentration of IL‐1α was quantified using a microBCA assay (Thermo Fisher Scientific) according to the manufacturer's instructions, and IL‐1α encapsulation efficiency and loading capacity were estimated. To assess in vitro release kinetics of IL‐1α, samples of IL‐1α‐MPs were suspended in PBS and incubated at 37°C and 300 rpm in an orbital incubator shaker. Aliquots of supernatant were collected at indicated time intervals and replaced with fresh PBS. IL‐1α released from microparticles was quantified using microBCA assay. The release data were expressed as the mean of cumulative IL‐1α released into PBS. Activity of the released IL‐1α from the IL‐1α‐MPs were tested in vitro, by culturing human PBMCs with IL‐1α ‐MPs at different time points and measuring interleukin‐6 (IL‐6) release in the culture media by enzyme‐linked immunosorbent assay (ELISA) using standard manufacturer protocols.

### 2.3 Cell lines and reagents

The UPCI:SCC152 (SCC152) cell line was purchased from American Type Culture Collection (ATCC, Manassas, VA, USA). The mEERL cell line (murine oropharyngeal epithelial cell line stably transformed with HPV E6 and E7 together with hRas and luciferase) was a gift from Dr Paola Vermeer (Department of Surgery, University of South Dakota Sanford School of Medicine, South Dakota, USA). SCC152 cell lines were cultured in Dulbecco's Modified Eagle's Medium (DMEM) containing 10% fetal bovine serum (FBS), 0.1% gentamicin, and 1% non‐essential amino acids. The mEERL cells were cultured in DMEM supplemented with 40.5% 1:1 DMEM/Hams F12, 10% FBS, 0.1% gentamicin, 0.005% hydrocortisone, 0.05% transferrin, 0.05% insulin, 0.0014% tri‐iodothyronine and 0.005% epidermal growth factor. Both the cell lines are adherent and were cultured in vented flasks at 37°C and 5% CO_2_ in a humidified incubator. Both cell lines were routinely checked for mycoplasma contamination and none of the cell lines were used beyond 10–12 passage after resuscitation of frozen aliquots. Dichloromethane, sucrose, and mowiol (poly(vinyl alcohol)) (PVA, MW ~ 67,000) were purchased from Sigma‐Aldrich (USA). The polyanhydride copolymer (CPH:SA 20:80) was provided by Dr. Aliasger Salem (Department of Pharmaceutical Sciences and Experimental Therapeutics, University of Iowa).

### 2.4 In vitro drug treatment and immune cell activation

Recombinant human IL‐1α (rIL‐1α) was purchased from BioLegend (San Diego, California) and used at a concentration of 10–100 ng/ml for 24 h. Human peripheral blood mononuclear cells (PBMCs) were collected from healthy donor blood (DeGowin Blood Center, University of Iowa Hospitals and Clinics) by density gradient centrifugation using Ficoll paque. SCC152 cells were co‐cultured with human PBMCs 1:3 in 96‐well plates. PBMCs were then stained with a cocktail of fluorochrome conjugated antibodies: CD45‐PE‐Cy5 (HI30, BioLegend), CD3‐PE‐Cy7 (HIT3a, BioLegend), CD19‐Pacific Blue (SJ25C1, BioLegend), CD4‐Alexa Fluor 594 (RPA‐T4, Biolegend), CD8‐PerCP (SK1, BioLegend), CD56‐APC (5.1H11, BioLegend), CD14‐PerCP‐Cyanine5.5 (63D3, BioLegend), CD11c‐BV421 (3.9, BioLegend), HLA‐DR‐APC‐Cy7 (L243, BioLegend), BDCA‐4‐PE (12C2, BioLegend), CD123‐Alexa Fluor 700 (6H6, BioLegend), CD40‐BV605 (5C3, BioLegend) and CD69‐FITC (FN50, BioLegend). After antibody staining and fixation, cells were acquired on a 5 laser Cytek Aurora Cytometer (Flow Cytometry Facility, University of Iowa, IA) and analyzed through FlowJo software (BD Biosciences). NK cells were gated as CD45+ CD3− CD19‐CD56+, T cells were defined as CD45+ CD3+ CD19‐CD4+/CD8+, monocytes were gated as CD45+ CD3− CD19−, CD56− HLA‐DR+ CD14+. pDCs were gated as CD45+ CD3− CD19−, CD56− HLA‐DR+ CD11c‐BDCA‐4+ CD123+ and mDCs were defined as CD45+ CD3− CD19− CD56− HLA‐DR+ CD11c−. CD69+ NK cells, CD69+ T cells, CD40+ pDCs, CD40+ monocytes, and CD40+ mDCs were considered activated. The gating strategies are shown in Figure S1. Percentage of positive cells were then quantified and plotted as fold change compared to control.

### 2.5 In vivo tumor cell implantation and drug treatment

Four‐six‐week‐old male C57BL/6J mice were purchased from The Jackson Laboratory. All experimental animals were housed in the Animal Care Facility at the University of Iowa and handled using aseptic procedures. Mice were allowed at least 5 days to acclimate prior to handling, and food and water were made freely available. All procedures were approved by the Institutional Animal Care and Use Committee (IACUC) at the University of Iowa and conformed to the guidelines established by the National Institutes of Health. The murine oropharyngeal epithelial cell line stably transformed with HPV E6 and E7 together with hRas and luciferase (mEERL) was a gift from Dr. Paola Vermeer (Department of Surgery, University of South Dakota Sanford School of Medicine). These cells (1 × 10^6^ cells/100 μg PBS/mouse) were inoculated subcutaneously into the right flank of each animal. When tumors became palpable (~2–4 mm in any direction) mice (*n* = 6 mice/treatment group) were treated i.p. with IL‐1α‐MPs (equivalent to 3.5 μg rIL‐1α in 29.4 mg MPs) or the corresponding blank MPs (29.4 mg) on Treatment Days 1 and 5. Saline (100 μl) and rIL‐1α (3.5 μg) was used as a negative and positive control respectively. For the dose scalation study mice (*n* = 10 mice/treatment group) were treated with a single dose of rIL‐1α or IL‐1α‐MPs (equivalent to 7.5 μg rIL‐1α/10.7 mg MPs or 15 μg rIL‐1α/21.4 mg MPs), blank MPs (21.4 mg) or saline (100 μl). Mice were evaluated daily, and weight and tumor measurements taken periodically using Vernier calipers. Tumor volumes were calculated using the formula: tumor volume = (length × width^2^)/2 where the length was the longest dimension, and width was the dimension perpendicular to length. Mice were euthanized via CO_2_ gas asphyxiation or lethal overdose of sodium pentobarbital (100 mg/kg) when tumor diameter exceeded 1.5 cm in any dimension.

### 2.6 Cytokine storm and toxicity assessments

Blood samples were taken from a subset (*n* = 3–4) of mice from each treatment group 24 h after the second treatment on Treatment Day 6. Serum was collected and analyzed for changes in proinflammatory cytokine levels using a mouse Bio‐Plex 23 panel assay kit as per the manufacturer's instructions (Bio‐Rad, Hercules, California, USA). A Bio‐Plex array reader and Bio‐Plex Manager software were used to quantify and calculate analyte concentrations. For acute and chronic toxicity assessment, blood samples were drawn on Treatment Days 2 and 30 from a subset (*n* = 4) of mice treated with two doses (3.5 μg each) of rIL‐1α or equivalent CPH:SA IL‐1α‐MPs for a total of 7.5 μg IL‐1α. Serum of both timepoints were tested for alanine aminotransferase (ALT), alkaline phosphatase (ALP), aspartate aminotransferase (AST), total bilirubin (TBIL), and creatinine levels (IDEXX BioAnalytics (Iowa City, IA)).

### 2.7 Analysis of tumor and lymph node immune cell infiltration

Tumors and draining lymph nodes (lymph nodes near tumor site) were harvested from a subset of mice (*n* = 3–4/treatment group). Harvested tumors (~100 mg tissue) and lymph nodes were mechanically dissociated (gentleMACS Dissociator; Miltenyi Biotec) into single‐cell suspensions. Red blood cells were lysed using RBC lysis buffer (Biolegend). Single cell suspensions were then filtered through 70‐μm mesh filters, washed with buffer, and incubated with viability dye for 30 min. Cells were washed and incubated with the murine antibodies: CD45‐Alexa Fluor700 (104, BioLegend), CD3e‐BUV737 (145‐2C11, BD Biosciences), CD4‐PerCP (GK1.5, BioLegend), CD8α‐APC‐Cy7 (QA17A07, BioLegend), CD11b‐PE‐Cy5 (M1/70, BioLegend), CD11c‐KIRAVIA Blue520 (N418, BioLegend), CD19‐BV785 (6D5, BioLegend), CD335‐BV605 (29A1.4, BioLegend), F4/80‐PE‐Cy7 (QA17A29, BioLegend), Ly‐6G‐PerCP‐Cy5.5 (1A8, BioLegend), Ly‐6C‐BV711(HK1.4, BioLegend), and MHC class II‐BUV563 (M5/114.15.2, BD Biosciences) for 30 min at 40°C. Mouse FcR blocker were used to block nonspecific binding of the marker antibody. Fixation/Permeabilization Solution kits (eBioscience) were used to stain intracellular cytokines. Cells were then stained with antibody against IFNγ (XMG1.2, Biolegend). HPV+ CD8+ T cells in lymph nodes were detected by HPV E7‐specific iTAg tetramer staining (PE‐H‐2Db HPV 16 E7 (RAHYNIVTF), MBL International Corporation). Cells were washed, resuspended in 2% paraformaldehyde in FACS buffer and acquired on a 5 laser Cytek Aurora Cytometer (Flow Cytometry Facility, University of Iowa, IA) and analyzed through FlowJo software (BD Biosciences). The number of tumor‐infiltrating immune cells was normalized by tumor weight.

### 2.8 Assessment of blood pressure in conscious mice by radiotelemetry

C57Bl/6 mice (*n* = 6 mice/treatment group) were anesthetized with ketamine (91 μg/g, IP) and xylazine (9.1 μg/g, IP) and radiotelemetry probes (PC10, DSI) that allowed for measurement of arterial blood pressure and heart rate were implanted into the thoracic aorta through the left common carotid artery, as described previously.^45^, ^46^ The mice were allowed a week to recover. Each animal was housed in individual cages in the Animal Care Facility and monitored daily for signs of ill‐health. Mice were then inoculated with HNSCC (mEERL) cells and treated with rIL‐1α and IL‐1α‐MPs (equivalent to 7.5 μg IL‐1α on Days 10) as already described above. Changes in blood pressure (mean arterial pressure) and heart rate (HR) were measured over the entire experimental period (18 days) at a sampling rate of 500 Hz for 10 s once every 5 min using the DSI Acquisition software.

### 2.9 Statistical analyses

Statistical analysis was carried out using GraphPad PrismV.8 for Windows (GraphPad Software, San Diego, California, USA). Differences between three or more groups were determined by one‐way analysis of variance (ANOVA) with Tukey post‐tests. Linear regression models were used to estimate the group‐specific change in tumor growth curves. Two‐way ANOVA followed by Tukey post‐tests comparison were used to compare difference in blood pressure in each day between groups. Heat maps were generated using ggplot2 of RStudio (r version 4.1.1). Statistical significance was defined as *p* < .05.

## 3 RESULTS

### 3.1 Interleukin‐1α stimulates immune cell activation

**FIGURE 1:**
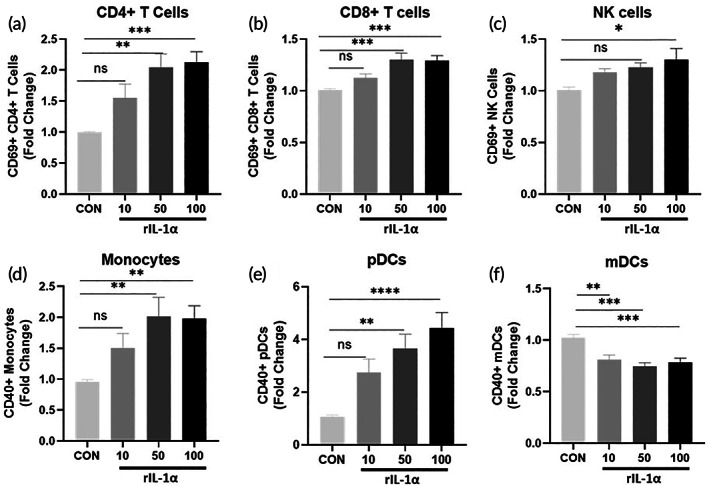
Interleukin‐1α stimulates immune cell activation. SCC152 cells were co‐cultured with human peripheral blood mononuclear cells (PBMCs) and treated with three doses (10, 50 and 100 ng/ml) of human recombinant IL‐1α (rIL‐1α) for 24 h. PBS was used as a control (CON). Activation of CD4+ (a) and CD8+ (b) T cells, natural killer (NK) cells (c) monocytes (d), plasmacytoid dendritic cells (pDCs) (e), and myeloid dendritic cells (mDCs) (f) was detected by flow cytometry. Bars represent the mean of *n* = 3 experiments. Average values were normalized to CON and plotted as fold change. Error bars represent standard error from the mean. *p < .05; **p < .01; ***p < .001; ns, nonsignificant.

To demonstrate the potential of IL‐1α to trigger anti‐tumor immune activity, human PBMCs were co‐cultured with SCC152 HNSCC cells and treated with PBS and three doses (10, 50 and 100 ng/ml) of human recombinant IL‐1α (rIL‐1α) for 24 h. Flow cytometric analysis showed that rIL‐1α significantly increased levels of activated (CD69+) CD4+ T cells (Figure 1a), CD8+ T cells (Figure 1b) and NK cells (Figure 1c); and activated (CD40+) monocytes (Figure 1d) and pDCs (Figure 1e). Dose dependency of activation was observed for CD4+ T cells (Figure 1a), monocytes (Figure 1d) and pDCs (Figure 1e). Maximum immune cell activation was observed at 50 ng/ml rIL‐1α. Additionally, pDCs appeared to be the most sensitive to rIL‐1α, with a nearly 4–5‐fold activation at the 100 ng/ml dose than the activation of the other immune cells analyzed (Figure 1e). Levels of activated mDCs were decreased with rIL‐1α treatment with no dose response observed (Figure 1f). Raw values (percent of population) of immune cells activated by IL‐1α are shown in Figure S1.

### 3.2 Characterization of interleukin‐1 alpha loaded microparticles

**FIGURE 2:**
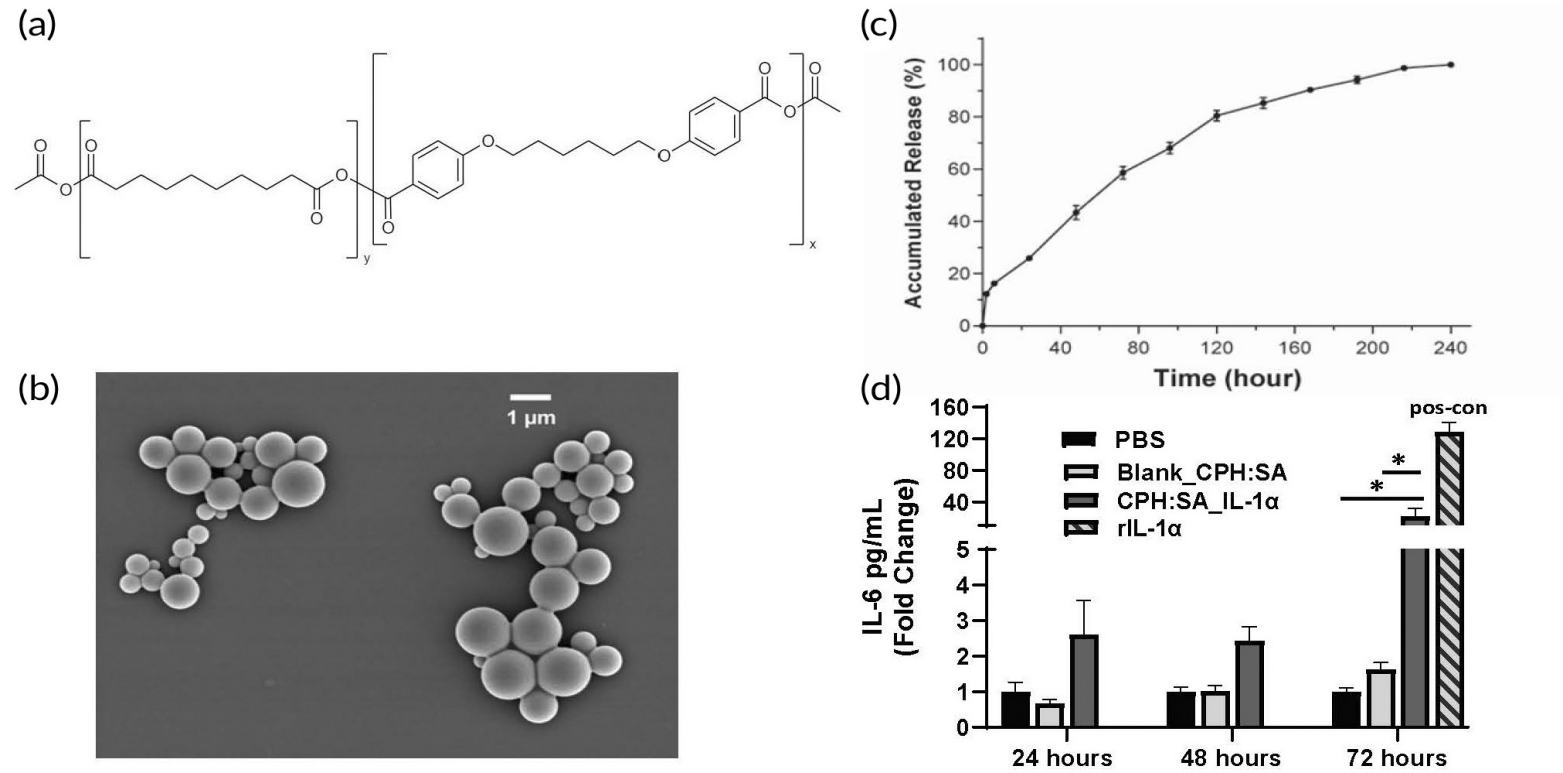
Characterization of IL‐1α loaded CPH:SA microparticles. (a) Chemical structure of CPH:SA microparticles (MPs) *x*:*y* (20:80). (b) Scanning electron micrograph of recombinant IL‐1α (rIL‐1α) loaded CPH:SA MPs (CPH:SA IL‐1α‐MPs). (c) In vitro release profile of CPH:SA IL‐1α‐MPs, *n* = 3. (d) In vitro activity test of CPH:SAIL‐1α‐MPs. Human peripheral blood mononuclear cells (PBMCs) were treated with 143 μg/ml CPH:SA IL‐1α‐MPs (equivalent to 100 ng/ml rIL‐1α) or Blank particles. Recombinant IL‐1α (50 ng/ml) was used as a positive control. Interleukin‐6 (IL‐6) release in the culture media were measured by ELISA. *n* ≥ 3. Data points are expressed as mean ± standard error. *p < .05.

CPH:SA MPs (20:80) loaded with IL‐1α (IL‐1α‐MPs) were synthesized and the average particle size, polydispersity index (PDI), and zeta potentials of IL‐1α loaded microparticles are shown in Table 1. Particles were prepared in two separated batches. Both batches of particles had similar size, zeta potential, and encapsulation efficiency except the loading capacity. The loading capacity between the two batches is different due to differences in IL‐1α protein used during particle preparation. The chemical structure of CPH:SA MPs are shown in Figure 2a. SEM images showed that the IL‐1α‐MPs possessed a spherical shape and smooth surface (Figure 2b). In vitro release kinetics demonstrated a sustained release profile in 0.1 M PBS for up to 10 days (Figure 2c). Release kinetics of the second batch particles showed no difference in release profile (Figure S2). Activity of released IL‐1α from the particles were tested by measuring IL‐1α‐induced IL‐6 release from PBMCs treated with CPH:SA IL‐1α‐MPs and respective positive (rIL‐1α) and negative controls (PBS and Blank_CPH:SA). CPH:SA IL‐1α‐MP treatment significantly increased IL‐6 secretion from PBMCs after 72 h treatment (Figure 2d). Empty particles did not induce IL‐6 release compared to the PBS control. This data suggests that encapsulation of IL‐1α does not suppress its biological activity.

### 3.3 CPH:SA IL‐1α‐MPs suppress IL‐1α‐induced weight loss and cytokine storm in mice

**FIGURE 3:**
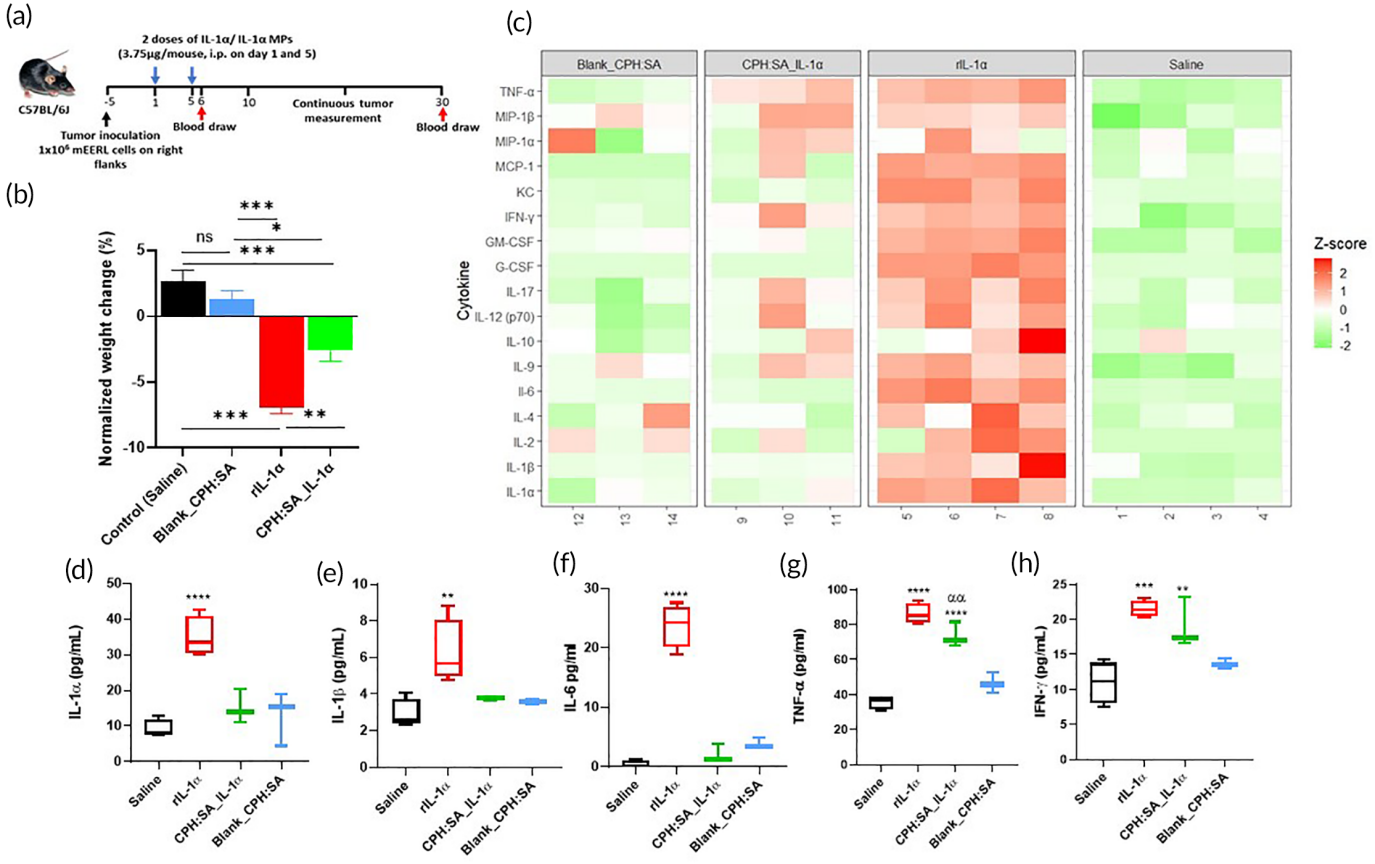
CPH:SA IL‐1α‐MPs suppresses weight loss in experimental animals. (a) HNSCC (mEERL) tumor‐bearing C57Bl/6 mice (*n* = 6/treatment group) were treated with CPH:SA IL‐1α‐MPs or rIL‐1α (3.75 μg) on Treatment Days 1 and 5 (total IL‐1α = 7.5 μg) intraperitoneally. (b) Average animal weights in each treatment group were plotted separately and expressed as percent weight loss on Treatment Day 6. (c) Blood was collected on Treatment Day 6 and pro‐inflammatory cytokines in serum were measured using a bead‐based multiplexed immunoassay system. Heat maps of all cytokines detected were plotted in (c). Upregulated cytokines are shown in red. Select pro‐inflammatory cytokine levels of IL‐1α (d), IL‐1β (e), IL‐6 (f), TNFα (g) and IFNγ (h) are shown. *p < .05; **p < .01; ***p < .001; ****p < .0001.

To begin to compare the effect of unencapsulated IL‐1α and IL‐1α‐MPs, male C57Bl/6 mice (*n* = 5–10 mice/treatment group) bearing mEERL HNSCC tumors were treated with saline, murine rIL‐1α, IL‐1α‐MPs (3.75 μg rIL‐1α/mouse, i.p) and corresponding Blank MPs on Treatment Days 1 and 5 (total = 7.5 μg IL‐1α, Figure 3a). Significant weight loss was observed in all rIL‐1α‐treated mice on Treatment Day 6 (Figure 3b). On the other hand, mice treated with IL‐1α‐MPs had significantly less weight reduction compared to rIL‐1α‐treated mice (Figure 3b). Circulating cytokines from a subset of mice from the aforementioned experiment were analyzed by multiplex cytokine assay 24 h of the last treatment (Treatment Day 6). Pro‐Inflammatory cytokines that were detectable are represented in the heatmap shown in Figure 3c. A strikingly different cytokine profile was observed between rIL‐1α and the rest of the treatment groups (Figure 3c). Treatment with rIL‐1α induced the expression of all pro‐inflammatory cytokines shown, whereas the cytokine profile of IL‐1α‐MPs were similar to the empty particle or the control group (Figure 3c). Additionally, rIL‐1α‐treated mice exhibited known signs of “cytokine storm” including elevated levels of IL‐1α (Figure 3d), IL‐1β (Figure 3e), IL‐6 (Figure 3f) and TNFα (Figure 3g) compared to control and Blank MP‐treated mice. Serum levels of IL‐9, G‐CSF, GM‐CSF, CXCL‐1, MIP‐1β and MCP‐1 were also significantly increased in rIL‐1α‐treated mice compared to control and Blank MP‐treated mice (Figure S3). On the other hand, IL‐1α‐MPs significantly increased only three inflammatory cytokines—TNFα (Figure 3g), MIP‐1β (Figure S3c) and IL‐9 (Figure S3f) compared to control and Blank MP‐treated mice. Lastly both rIL‐1α and IL‐1α‐MPs significantly increased IFNγ, which is a prominent cytokine in anti‐tumor immunity compared to control and Blank MP‐treated mice (Figure 3h). These results suggest that CPH:SA IL‐1α‐MPs may suppress the signs of cytokine storm induced by rIL‐1α. To determine if IL‐1α‐MPs produce any acute and chronic liver or kidney toxicity we measured serum biomarkers (ALP, AST, ALT, TBIL and creatine) at Treatment Day 6 and Treatment Day 30 (Figure S4). At Treatment Day 6, serum ALT levels were significantly lower with rIL‐1α and IL‐1α‐MP treatment compared to control and corresponding Blank MPs. In the case of serum creatinine levels, significant differences were observed only in rIL‐1α treatment compared to control and Blank MPs. At Treatment Day 30, there were no significant differences observed in serum biomarkers among the treatment groups. These results suggest that IL‐1α‐MPs may produce negligible acute or chronic toxicity in mice compared to the unencapsulated free cytokine.

### 3.4 Systemic bolus dose of CPH:SA_IL‐1α MPs are safe in HNSCC tumor bearing mice

**FIGURE 4:**
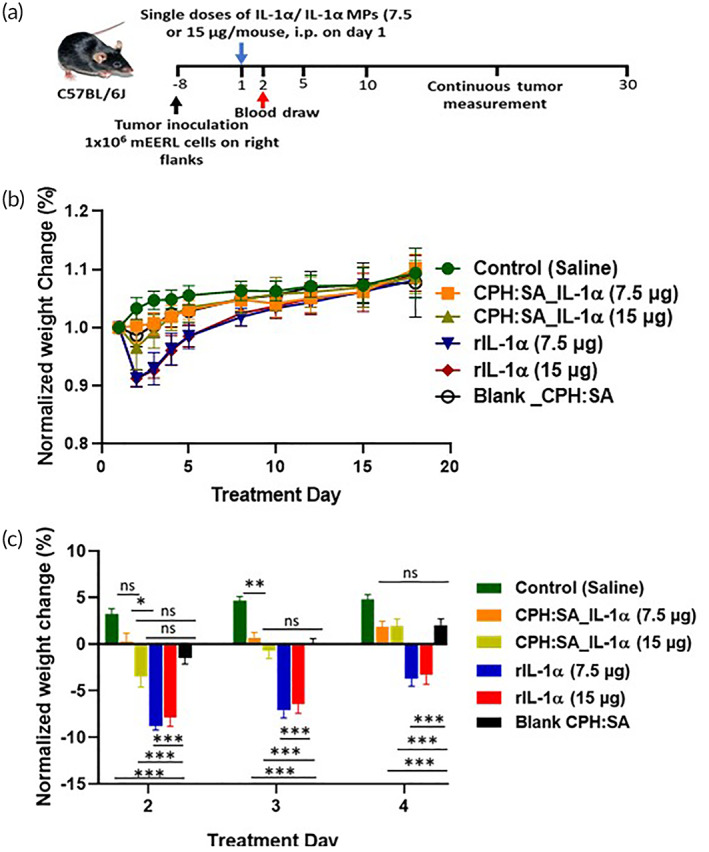
Bolus doses of CPH:SA IL‐1α MPs appear safe in HNSCC tumor‐bearing mice. (a) Single bolus doses of CPH:SA IL‐1α‐MPs or rIL‐1α (7.5 μg or 15 μg) were administered to mEERL tumor‐bearing C57Bl/6 mice (*n* = 9–10/treatment group) intraperitoneally. (b) Average changes in animal weight ‐ plotted as percent weight change were monitored over time. (c) Average animal weights in each treatment group were plotted separately and expressed as percent weight loss on Treatment Days 2, 3 and 4. Blank CPH:SA and saline were used as negative controls for both studies. Error bars represent SE from the mean. *p < .05; **p < .01.

To determine if higher doses of IL‐1α‐MPs would potentially be safe, a single dose of rIL‐1α or IL‐1α‐MPs (7.5 and 15 μg, i.p.) was administered to tumor bearing mice (Figure 4a). Here we used a separate batch of IL‐1α‐MPs with higher loading capacity. MP characteristics of this batch are shown in Table 1. Saline and Blank MPs were again used as controls. Both doses of rIL‐1α triggered identical weight loss in mice compared to saline and Blank MPs with recovery to baseline by Treatment Day 8 (Figure 4b). On the other hand, both IL‐1α‐MP doses produced minimal weight loss at Treatment Day 2, with the 15 μg dose triggering around a 3% initial weight loss which recovered by Treatment Days 3 and 4 (Figure 4b,c). These results indicate that CPH:SA MPs may provide an avenue to deliver large bolus doses of IL‐1α with minimal side effects.

### 3.5 IL‐1α evoked hypotension is prevented by CPH:SA IL‐1α‐MPs

**FIGURE 5:**
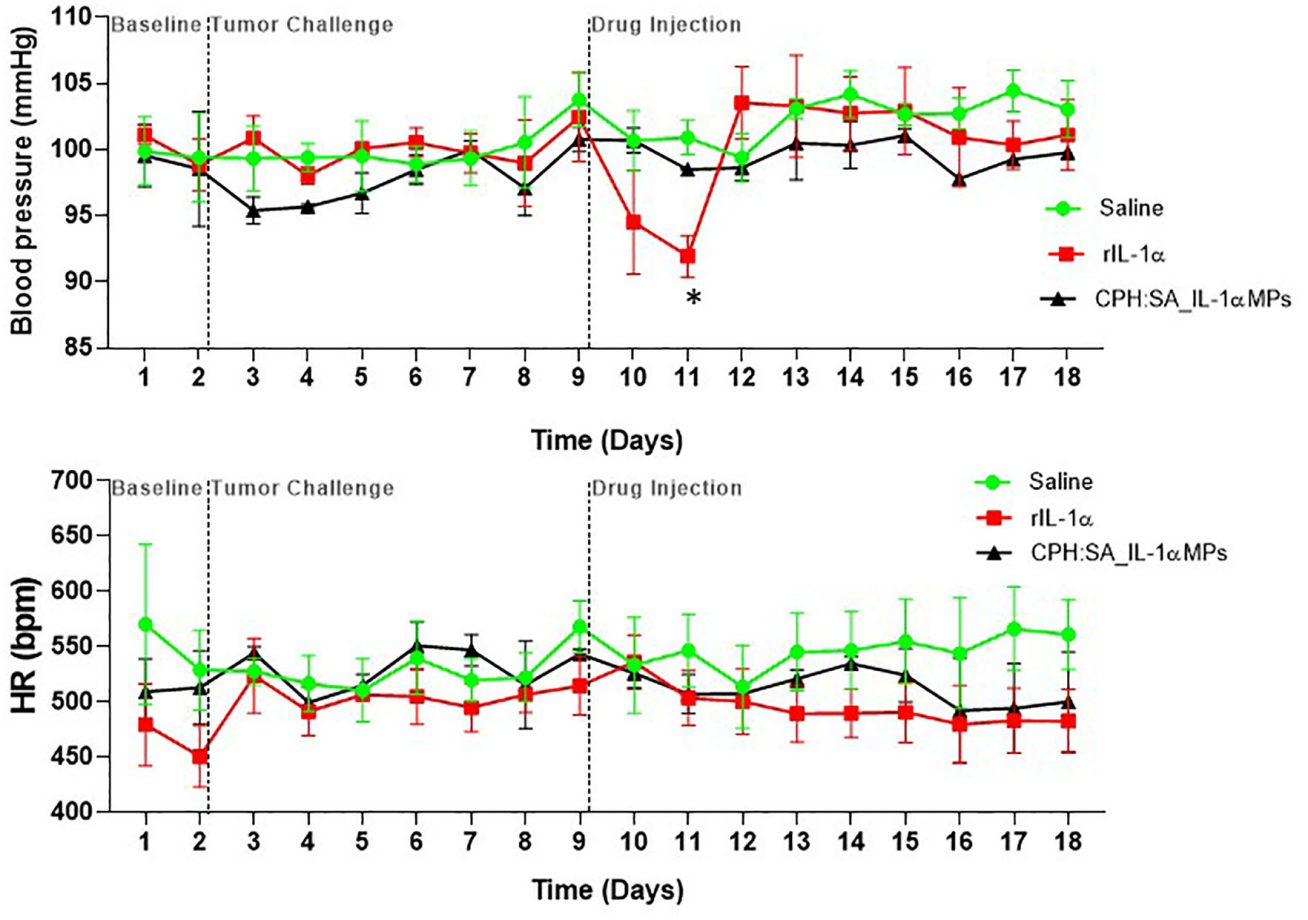
CPH:SA IL‐1α‐MPs prevents rIL‐1α‐induced hypotension. Single bolus doses of CPH:SA IL‐1α‐MPs or rIL‐1α (7.5 μg) were administered to mEERL tumor‐bearing C57Bl/6 mice (*n* = 9–10/treatment group) intraperitoneally. Blank_CPH:SA and saline were used as negative controls. Blood pressure (mean arterial pressure) (a) and heart rate (b) were measured over the experimental period. Data points represent the average of *n* = 3 mice/treatment group. Error bars represent standard error from the mean. *p < .05 versus saline and CPH:SA IL‐1α‐MPs.

One of the adverse effects of various cytokine therapies is the marked reduction in blood pressure. Therefore, we measured blood pressure and heart rate in a separate cohort of tumor‐bearing conscious mice before and after cytokine delivery via radiotelemetry. Mean arterial pressure reduced significantly in mice treated with rIL‐1α and recovered by Day 3, whereas mice that received an identical dose of rIL‐1α via CPH:SA IL‐1α‐MPs showed no changes in mean arterial pressure compared to control (Figure 5a). There were no significant changes in heart rate among the experimental groups (Figure 5b). These results suggest that CPH:SA IL‐1α‐MPs prevent rIL‐1α‐induced hypotension.

### 3.6 CPH:SA‐IL‐1α‐MPs exhibits similar tumor control compared to parent rIL‐1α molecule

**FIGURE 6:**
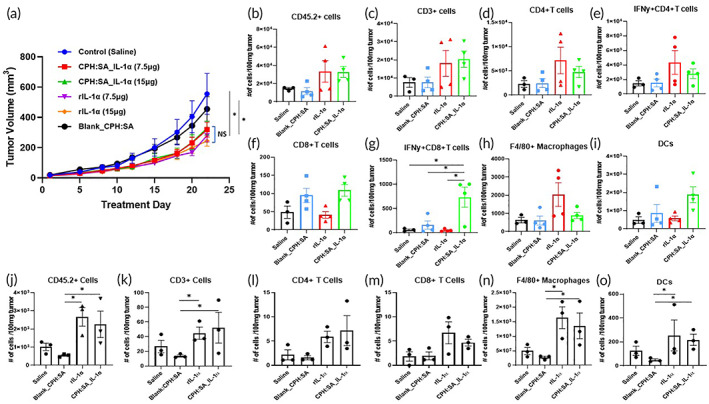
CPH:SA IL‐1α‐MPs exhibit similar tumor control compared to parent rIL‐1α. (a) HNSCC (mEERL) tumor‐bearing C57Bl/6 mice (*n* = 9–10 mice/treatment group) were treated with a single dose (7.5 μg or 15 μg) of rIL‐1α or equivalent CPH:SA IL‐1α‐MPs intraperitonially on Treatment Day 1 and tumor growth was monitored over time as in Figure 4a. Saline and Blank CPH:SA MPs were used as controls. Error bars represent standard error from the mean. Tumor growth curves within the bracket “]” were not significant (ns) from each other. *p < .05. (b–o) Tumors were harvested from a subset of HNSCC (mEERL) tumor‐bearing C57Bl/6 mice (*n* = 3/treatment group) treated with 7.5 μg (b–i) or 15 μg (j–o) of rIL‐1α or equivalent CPH:SA IL‐1α‐MPs intraperitonially on Treatment Day 1. Saline and Blank_CPH:SA MPs were used as controls. Tumor homogenates from the 7.5 μg dosing scheme were analyzed by flow cytometry for select immune subsets such as CD45.2+ (b), CD3+ (c), CD4+ (d) IFNγ+ CD4+ (e), CD8+ (f) and IFNγ+ CD8+ (g) T cells, macrophages (h) and dendritic cells (DCs) (i). Tumor homogenates from the 15 μg dosing scheme were also analyzed for select immune subsets such as CD45+ cells (j), CD3+ (k), CD4+ (l) CD8+ (m) T cells, macrophages (n) and DCs (o). Error bars represent standard error from the mean. *p < .05; **p < .01.

Observing the favorable safety profile we then compared the anti‐tumor effects of CPH:SA‐IL‐1α‐MPs with unencapsulated rIL‐1α according to Figure 4a. We found that IL‐1α treatment whether in free form or encapsulated all exhibited similar tumor growth inhibition compared to Blank‐MPs and saline controls (Figure 6a), suggesting that the use of CPH:SA MPs did not significantly suppress the anti‐tumor effects of IL‐1α. However, this data does raise additional questions about the lack of observed dose response of the 2 doses of IL‐1α. Nevertheless, these data suggest that the use of CPH:SA MPs did not significantly suppress the anti‐tumor effects of IL‐1α and that further manipulation of the MP dose and dosing schedule may yield significant and durable anti‐tumor effects.

### 3.7 CPH:SA IL‐1α‐MPs triggers the recruitment of tumor‐infiltrating immune cells

To investigate whether IL‐1α‐MPs would increase the recruitment of tumor infiltrating immune cells, tumors were harvested at Treatment Day 4 from a subset of mice (*n* = 3–4 mice/group) from saline, Blank MP, rIL‐1α (7.5 μg), and CPH:SA IL‐1α‐MPs (7.5 μg) treatment groups. Flow cytometric analysis revealed that both rIL‐1α and IL‐1α‐MPs generally increased most of the tumor immune cell populations analyzed compared to saline and Blank MP treatment (Figure 6b–i). However, significant differences were only observed for IFNγ+ CD8 T cells, which were high in CPH:SA IL‐1α‐MPs treatment compared to all other treatment (Figure 6g). We have also, analyzed the immune cell population in tumor after 22 days of the treatment to see if CPH:SA IL‐1αMP can produce sustained immune cell infiltration in the tumor micro‐environment. We found that CD45.2+ (Figure 6j), CD3+ (Figure 6k), macrophages (Figure 6n) and DCs (Figure 6o) where significantly increased in both rIL‐1α and IL‐1α MPs treatments compared to Blank MPs. These results again support the idea that CPH:SA MPs do not reduce the ability of IL‐1α to trigger a potential anti‐tumor immune response.

### 3.8 CPH:SA_IL‐1αMPs increases immune cell infiltration to the draining LN

**FIGURE 7:**
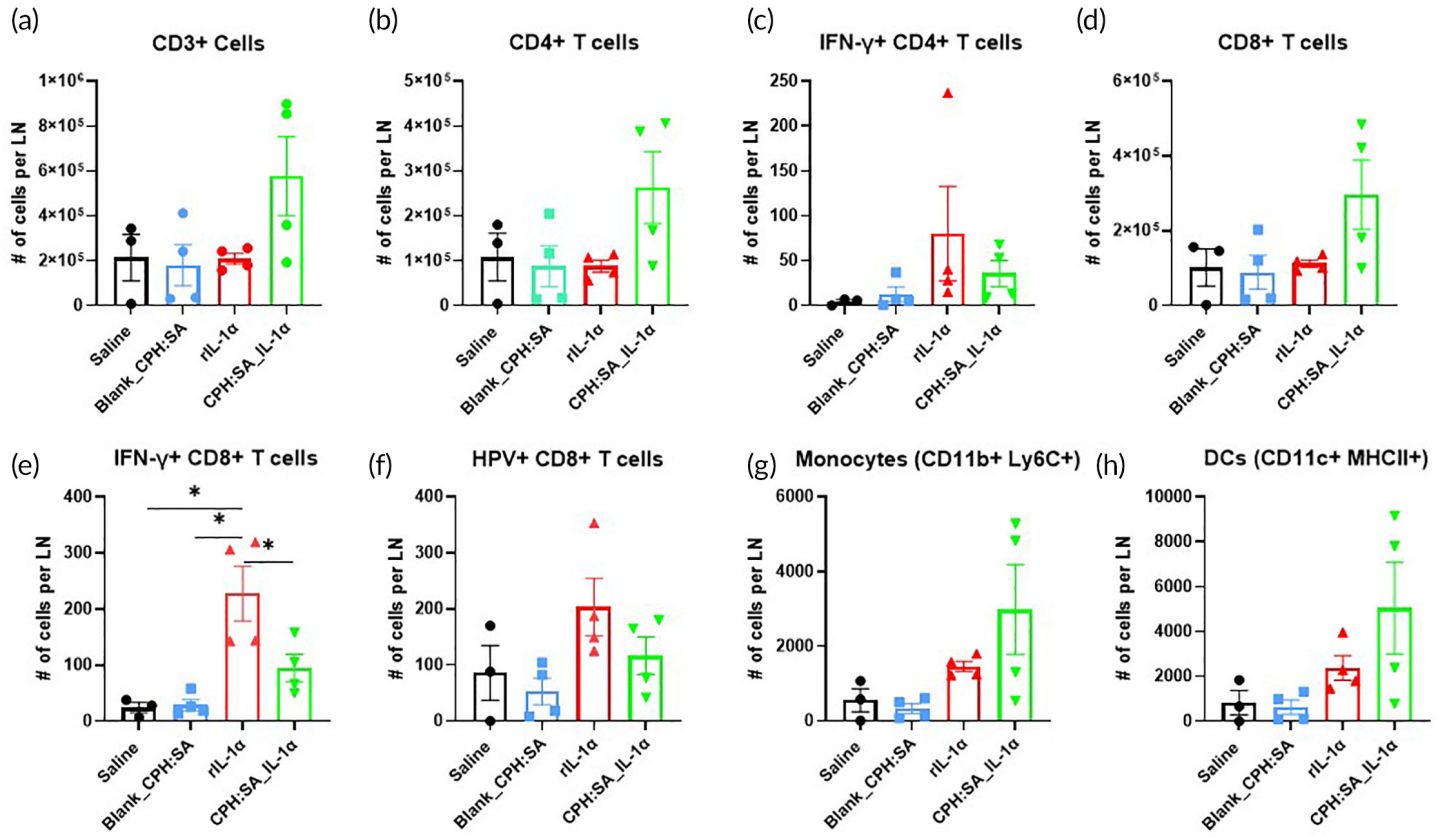
Systemic interleukin‐1 alpha (IL‐1α) treatment increases immune cell infiltration to the draining lymph node (LN). LNs were harvested from a subset of HNSCC (mEERL) tumor‐bearing C57Bl/6 mice (*n* = 3/treatment group) treated with 15 μg of rIL‐1α or equivalent CPH:SA IL‐1α‐MPs intraperitonially on Treatment Day 1 as in Figure 4a. Saline and Blank_CPH:SA MPs were used as controls. LN homogenates were analyzed by flow cytometry for select immune subsets such as CD3+ (a), CD4+ (b), IFNγ + CD4+ (c), CD8+ (d), IFNγ + CD8+ (e), and HPV+ CD8+ (f) T cells, monocytes (g) and dendritic cells (DCs) (h). Error bars represent standard error from the mean. *p < .05.

To assess systemic anti‐tumor immunity induced by CPH:SA IL‐1α‐MPs, lingual draining lymph nodes (LNs) at the tumor site were harvested from a subset of mice (*n* = 3–4 mice/group) at Treatment Day 4. LNs were stained and analyzed by flow cytometry for differences in immune cell subsets among the treatment groups (Figure 7a–h). In draining LNs, there was a general increase in CD3+ cells (Figure 7a), CD4+ T cells (Figure 7b), CD8+ T cells (Figure 7d), monocyte (Figure 7g) and DCs (Figure 7h) in CPH:SA IL‐1α‐MPs treatment compared to all other treatments (Saline, rIL‐1α and Blank_CPH:SA MPs). Although, CD3+ cells, CD4+ T cells, CD8+ T cells in rIL‐1α treated group were not higher compared to saline and Blank_CPH:SA MPs, there were significantly higher IFNγ+ CD8+ T cells (Figure 7e), which explains the observed anti‐tumor response in rIL‐1α treated mice. All these results suggest that the microparticle formulation of rIL‐1α does not alter the immunogenicity of parent rIL‐1α molecules. We also collected lymph node from subset of mice after 22 days of treatment and analyzed selected immune cell subset but did not find any difference in immune cell populations among the treatment groups (data not shown).

## 4 DISCUSSION

Here we show that CPH:SA IL‐1α‐MPs are potential safe alternatives to rIL‐1α delivery for cancer therapy. Although further pharmacokinetic studies are needed for a more detailed examination of in vivo release kinetics, we believe that the combination of our in vitro release kinetics data, in vivo cytokine results, telemetry and weight loss data all support a more favorable IL‐1α release profile into circulation when encapsulated in CPH:SA particles compared to free parent rIL‐1α. Additionally, encapsulation of IL‐1α into CPH:SA MPs did not reduce the bioactivity of IL‐1α nor the in vivo anti‐tumor immune response which are all promising indicators for further study as an acceptable cancer immunotherapeutic strategy. Our previous published work in this area demonstrated highly promising results using rIL‐1α encapsulated in 20:80 1,8‐bis(p‐carboxyphenoxy)‐3,6‐dioxaoctane (CPTEG): 1,6‐bis(p‐carboxyphenoxy) hexane (CPH) nanoparticles (NPs).^47^ Administration of a single dose of these NPs (equivalent to 7.5 μg IL‐1α) i.p. on Day 1 of treatment to breast tumor (TUBO)‐bearing BALB/c mice caused significant tumor regression in 80% of treated mice.^47^ However, significant weight loss was observed in the IL‐1α‐NP‐treated mice (compared to the empty NP‐treated mice) in the initial 5 days after treatment,^47^ which was likely due to undesirable particle burst release kinetics with 90% of IL‐1α being released in the first hour. Nevertheless, the observed anti‐tumor activity of these NPs led to the further identification of particle formulations with improved release kinetics and toxicity profiles.

As shown here, CPH:SA‐based IL‐1α‐MPs showed a sustained release profile accompanied by minimal weight loss. There are many reasons for the difference in release kinetics between the CPH:SA‐based IL‐1α‐MPs and the previously studied CPTEG:CPH IL‐1α‐NPs which include the size difference between MP and NP systems, different preparation procedures, and differences in interaction/packaging between rIL‐1α and polymer. However, CPH:SA polymers are more hydrophilic than CPTEG:CPH polymers and are likely more compatible for loading with water soluble cytokines^40^ which may explain the differences in release kinetics between these three polymers.

PLGA polymers as a delivery vehicle for IL‐1α have been previously tested.^48^ Unlike polyanhydrides, PLGA polymers undergo bulk erosion and not surface erosion.^49^ Therefore, these polymers do not show well‐defined drug release kinetics, particularly for water‐labile low molecular weight agents like cytokines/chemokines.^49^ PLGA‐based IL‐1α‐MPs demonstrated various degrees of burst release kinetics^48^ which supports our previous work with PLGA particles (data not shown). The stability of cytokines and other water‐labile drugs may also be compromised in PLGA polymers due to interaction with water before release.^49^ Indeed there was some loss of IL‐1α activity after release from PLGA microspheres during the in vitro release studies.^48^ Nevertheless, PLGA IL‐1α‐MP administration (equivalent to 0.2 μg/mouse) to NFS/N mice bearing malignant fibrosarcoma tumors showed significantly longer survival rates compared to experimental controls although no toxicity end points were analyzed in vivo.^48^

In addition to the sustained release kinetics, the low toxicity profile of the MPs in the present studies may be attributed to the circulating cytokine profile of the mice. As expected, rIL‐1α administration induced the release of a large number of proinflammatory cytokines in the blood stream (Figure 3c). This massive influx of inflammatory cytokines is known as cytokine release syndrome (CRS),^50^ which is commonly observed for certain types of immunotherapies that limits its use in various clinical settings.^6^, ^51^, ^52^ CRS can lead to the development of symptoms ranging from mild, flu‐like symptoms to severe life‐threatening events.^53^ One of the life threating conditions that can arise from CRS is hypotension, which is of high concern for cytokines like IL‐1α.^17^ All clinical studies reported hypotension from rIl‐1α and rIL‐1β therapy resulting in patient withdrawal from clinical trials and early termination of the trials. Given the lack of cytokine storm (Figure 3c) and hypotension (Figure 5a) observed in IL‐1α‐MP‐treated mice, it is worth following up on these studies using multiple doses and dosing schedules to confirm.

Lastly, it was surprising that that the three different dosing strategies of rIL‐1α tested here (3.75 μg × 2 doses, 7.5 μg once and 15 μg once) showed no major differences in weight loss (Figures 3b and 4). This may be due to the rapid induction of the interleukin‐1 receptor agonist (IL‐1RA) which blocks the binding of IL‐1 ligands to the IL‐1R1 and serves as a regulatory mechanism to control excessive inflammation. It is possible that the induction of IL‐1RA expression may also be responsible for the limited tumor growth delay. Alternatively, increased circulating rIL‐1α may be recruiting myeloid‐derived suppressor cells (MDSCs) to the tumor microenvironment which would suppress any anti‐tumor immune responses. Although we show that rIL‐1α can increase recruitment of immune cells in vivo involved in anti‐tumor immune responses (Figures 6 and 7), IL‐1 ligands and other cytokines can trigger the expansion, accumulation and function of MDSCs resulting in the expansion of Tregs and direct suppression of immune cells.^54^ Future work will investigate the aforementioned mechanistic theories with a goal of determining the effect of the MPs on anti‐tumor immune stimulation and immune suppression.

### 4.1 Conclusions

Altogether, CPH:SA‐based IL‐1α‐MPs demonstrated promising sustained systemic release kinetics accompanied by minimal weight loss, cytokine storm and hypotension and retention of anti‐tumor activity. Therefore, MPs based on CPH:SA formulations may be promising as delivery vehicle for IL‐1α and other cytokines to achieve safe, effective anti‐tumor responses for cancer patients.

## AUTHOR CONTRIBUTIONS

**M. M. Hasibuzzaman:** Conceptualization (equal); data curation (lead); formal analysis (lead); investigation (lead); methodology (supporting); visualization (lead); writing – original draft (lead); writing – review and editing (lead). **Rui He:** Data curation (supporting); investigation (supporting); methodology (supporting); writing – review and editing (supporting). **Ishrat Nourin Khan:** Investigation (supporting); methodology (supporting); writing – review and editing (supporting). **Rasna Sabharwal:** Methodology (supporting); resources (supporting); software (supporting); supervision (supporting); visualization (supporting); writing – review and editing (supporting). **Aliasger Salem:** Conceptualization (supporting); methodology (equal); resources (supporting); supervision (equal); writing – original draft (supporting); writing – review and editing (supporting). **Andrean Llewela Simons‐Burnett:** Conceptualization (lead); data curation (supporting); formal analysis (supporting); funding acquisition (lead); methodology (lead); project administration (lead); resources (lead); supervision (lead); writing – original draft (supporting); writing – review and editing (lead).

## CONFLICT OF INTERESTS

No conflicts of interest to disclose.

### PEER REVIEW

The peer review history for this article is available at https://publons.com/publon/10.1002/btm2.10465.

## Supporting information


**Figure S1:** Supporting information.

**Figure S2:** Supporting information.

**Figure S3:** Supporting information.

**Figure S4:** Supporting information.

## Data Availability

The data that support the findings of this study are available from the corresponding author upon reasonable request.

## References

[1] Chen DS , Mellman I . Oncology meets immunology: the cancer‐immunity cycle. Immunity. 2013;39(1):1‐10. 23890059 10.1016/j.immuni.2013.07.012

[2] Chen DS , Mellman I . Elements of cancer immunity and the cancer‐immune set point. Nature. 2017;541(7637):321‐330. 28102259 10.1038/nature21349

[3] Gresser I , Bourali C . Antitumor effects of interferon preparations in mice. J Natl Cancer Inst. 1970;45(2):365‐376. 5514991

[4] Golomb HM , Jacobs A , Fefer A , et al. Alpha‐2 interferon therapy of hairy‐cell leukemia: a multicenter study of 64 patients. J Clin Oncol. 1986;4(6):900‐905. 3519880 10.1200/JCO.1986.4.6.900

[5] Solal‐Celigny P , Lepage E , Brousse N , et al. Recombinant interferon alfa‐2b combined with a regimen containing doxorubicin in patients with advanced follicular lymphoma. Groupe d'Etude des Lymphomes de l'Adulte. N Engl J Med. 1993;329(22):1608‐1614. 8232429 10.1056/NEJM199311253292203

[6] Berraondo P , Sanmamed MF , Ochoa MC , et al. Cytokines in clinical cancer immunotherapy. Br J Cancer. 2019;120(1):6‐15. 30413827 10.1038/s41416-018-0328-y PMC6325155

[7] Fyfe GA , Fisher RI , Rosenberg SA , Sznol M , Parkinson DR , Louie AC . Long‐term response data for 255 patients with metastatic renal cell carcinoma treated with high‐dose recombinant interleukin‐2 therapy. J Clin Oncol. 1996;14(8):2410‐2411. 8708739 10.1200/JCO.1996.14.8.2410

[8] Gutterman JU , Blumenschein GR , Alexanian R , et al. Leukocyte interferon‐induced tumor regression in human metastatic breast cancer, multiple myeloma, and malignant lymphoma. Ann Intern Med. 1980;93(3):399‐406. 6159812 10.7326/0003-4819-93-3-399

[9] Kirkwood JM , Ernstoff MS . Interferons in the treatment of human cancer. J Clin Oncol. 1984;2(4):336‐352. 6323641 10.1200/JCO.1984.2.4.336

[10] Marth C , Windbichler GH , Hausmaninger H , et al. Interferon‐gamma in combination with carboplatin and paclitaxel as a safe and effective first‐line treatment option for advanced ovarian cancer: results of a phase I/II study. Int J Gynecol Cancer. 2006;16(4):1522‐1528. 16884360 10.1111/j.1525-1438.2006.00622.x

[11] Windbichler GH , Hausmaninger H , Stummvoll W , et al. Interferon‐gamma in the first‐line therapy of ovarian cancer: a randomized phase III trial. Br J Cancer. 2000;82(6):1138‐1144. 10735496 10.1054/bjoc.1999.1053 PMC2363351

[12] Atkins MB , Lotze MT , Dutcher JP , et al. High‐dose recombinant interleukin 2 therapy for patients with metastatic melanoma: analysis of 270 patients treated between 1985 and 1993. J Clin Oncol. 1999;17(7):2105‐2116. 10561265 10.1200/JCO.1999.17.7.2105

[13] Fyfe G , Fisher RI , Rosenberg SA , Sznol M , Parkinson DR , Louie AC . Results of treatment of 255 patients with metastatic renal cell carcinoma who received high‐dose recombinant interleukin‐2 therapy. J Clin Oncol. 1995;13(3):688‐696. 7884429 10.1200/JCO.1995.13.3.688

[14] Kammula US , White DE , Rosenberg SA . Trends in the safety of high dose bolus interleukin‐2 administration in patients with metastatic cancer. Cancer. 1998;83(4):797‐805. 9708948

[15] Lee DS , White DE , Hurst R , Rosenberg SA , Yang JC . Patterns of relapse and response to retreatment in patients with metastatic melanoma or renal cell carcinoma who responded to interleukin‐2‐based immunotherapy. Cancer J Sci Am. 1998;4(2):86‐93. 9532410

[16] Rosenberg SA , Yang JC , White DE , Steinberg SM . Durability of complete responses in patients with metastatic cancer treated with high‐dose interleukin‐2: identification of the antigens mediating response. Ann Surg. 1998;228(3):307‐319. 9742914 10.1097/00000658-199809000-00004 PMC1191483

[17] Veltri S , Smith JW 2nd . Interleukin 1 trials in cancer patients: a review of the toxicity, antitumor and hematopoietic effects. Oncologist. 1996;1(4):190‐200. 10387988

[18] Dinarello CA . Overview of the interleukin‐1 family of ligands and receptors. Semin Immunol. 2013;25(6):389‐393. 24275600 10.1016/j.smim.2013.10.001

[19] Dinarello CA . Interleukin‐1 in the pathogenesis and treatment of inflammatory diseases. Blood. 2011;117(14):3720‐3732. 21304099 10.1182/blood-2010-07-273417 PMC3083294

[20] Dinarello CA , Conti P , Mier JW . Effects of human interleukin‐1 on natural killer cell activity: is fever a host defense mechanism for tumor killing? Yale J Biol Med. 1986;59(2):97‐106. 3488622 PMC2590122

[21] Eisenthal A , Rosenberg SA . The effect of various cytokines on the in vitro induction of antibody‐dependent cellular cytotoxicity in murine cells. Enhancement of IL‐2‐induced antibody‐dependent cellular cytotoxicity activity by IL‐1 and tumor necrosis factor‐alpha. J Immunol. 1989;142(7):2307‐2313. 2647849

[22] Fujiwara T , Grimm EA . Regulation of lymphokine‐activated killer cell induction by human recombinant IL‐1 receptor antagonist. Obligate paracrine pathway of IL‐1 during lymphokine‐activated killer cell induction. J Immunol. 1992;148(9):2941‐2946. 1374105

[23] Herman J , Dinarello CA , Kew MC , Rabson AR . The role of interleukin 1 (IL 1) in tumor‐NK cell interactions: correction of defective NK cell activity in cancer patients by treating target cells with IL 1. J Immunol. 1985;135(4):2882‐2886. 2993420

[24] Pullyblank AM , Guillou PJ , Monson JR . Interleukin 1 and tumour necrosis factor alpha may be responsible for the lytic mechanism during anti‐tumour antibody‐dependent cell‐mediated cytotoxicity. Br J Cancer. 1995;72(3):601‐606. 7669568 10.1038/bjc.1995.380 PMC2033875

[25] Ben‐Sasson SZ , Caucheteux S , Crank M , Hu‐Li J , Paul WE . IL‐1 acts on T cells to enhance the magnitude of in vivo immune responses. Cytokine. 2011;56(1):122‐125. 21843950 10.1016/j.cyto.2011.07.006 PMC3171626

[26] Ben‐Sasson SZ , Hogg A , Hu‐Li J , et al. IL‐1 enhances expansion, effector function, tissue localization, and memory response of antigen‐specific CD8 T cells. J Exp Med. 2013;210(3):491‐502. 23460726 10.1084/jem.20122006 PMC3600912

[27] Ben‐Sasson SZ , Hu‐Li J , Quiel J , et al. IL‐1 acts directly on CD4 T cells to enhance their antigen‐driven expansion and differentiation. Proc Natl Acad Sci U S A. 2009;106(17):7119‐7124. 19359475 10.1073/pnas.0902745106 PMC2678417

[28] Haabeth OA , Lorvik KB , Yagita H , Bogen B , Corthay A . Interleukin‐1 is required for cancer eradication mediated by tumor‐specific Th1 cells. Onco Targets Ther. 2016;5(1):e1039763. 10.1080/2162402X.2015.1039763 PMC4760324 26942052

[29] Belardelli F , Ciolli V , Testa U , et al. Anti‐tumor effects of interleukin‐2 and interleukin‐1 in mice transplanted with different syngeneic tumors. Int J Cancer. 1989;44(6):1108‐1116. 2606579 10.1002/ijc.2910440629

[30] Braunschweiger PG , Basrur VS , Cameron D , et al. Modulation of cisPlatin cytotoxicity by interleukin‐1 alpha and resident tumor macrophages. Biotherapy. 1997;10(2):129‐137. 9373735 10.1007/BF02678540

[31] Nakamura S , Nakata K , Kashimoto S , Yoshida H , Yamada M . Antitumor effect of recombinant human interleukin 1 alpha against murine syngeneic tumors. Jpn J Cancer Res. 1986;77(8):767‐773. 3093425

[32] Cooper MA , Fehniger TA , Ponnappan A , Mehta V , Wewers MD , Caligiuri MA . Interleukin‐1beta costimulates interferon‐gamma production by human natural killer cells. Eur J Immunol. 2001;31(3):792‐801. 11241284 10.1002/1521-4141(200103)31:3<792::aid-immu792>3.0.co;2-u

[33] Haabeth OA , Bogen B , Corthay A . A model for cancer‐suppressive inflammation. Onco Targets Ther. 2012;1(7):1146‐1155. 10.4161/onci.21542 PMC3494627 23170261

[34] Sugita S , Kawazoe Y , Imai A , et al. Mature dendritic cell suppression by IL‐1 receptor antagonist on retinal pigment epithelium cells. Invest Ophthalmol Vis Sci. 2013;54(5):3240‐3249. 23532521 10.1167/iovs.12-11483

[35] Eriksson U , Kurrer MO , Sonderegger I , et al. Activation of dendritic cells through the interleukin 1 receptor 1 is critical for the induction of autoimmune myocarditis. J Exp Med. 2003;197(3):323‐331. 12566416 10.1084/jem.20021788 PMC2193833

[36] Wesa AK , Galy A . IL‐1 beta induces dendritic cells to produce IL‐12. Int Immunol. 2001;13(8):1053‐1061. 11470775 10.1093/intimm/13.8.1053

[37] Aarreberg LD , Wilkins C , Ramos HJ , et al. Interleukin‐1beta signaling in dendritic cells induces antiviral interferon responses. MBio. 2018;9(2):00342‐18. 10.1128/mBio.00342-18 PMC5874908 29559569

[38] Yang F , Shi K , Jia YP , Hao Y , Peng JR , Qian ZY . Advanced biomaterials for cancer immunotherapy. Acta Pharmacol Sin. 2020;41(7):911‐927. 32123302 10.1038/s41401-020-0372-z PMC7468530

[39] Koerner J , Horvath D , Herrmann VL , et al. PLGA‐particle vaccine carrying TLR3/RIG‐I ligand Riboxxim synergizes with immune checkpoint blockade for effective anti‐cancer immunotherapy. Nat Commun. 2021;12(1):2935. 34006895 10.1038/s41467-021-23244-3 PMC8131648

[40] Wafa EI , Geary SM , Goodman JT , Narasimhan B , Salem AK . The effect of polyanhydride chemistry in particle‐based cancer vaccines on the magnitude of the anti‐tumor immune response. Acta Biomater. 2017;50:417‐427. 28063991 10.1016/j.actbio.2017.01.005 PMC5316298

[41] Geary SM , Hu Q , Joshi VB , Bowden NB , Salem AK . Diaminosulfide based polymer microparticles as cancer vaccine delivery systems. J Control Release. 2015;220(Pt B):682‐690. 26359124 10.1016/j.jconrel.2015.09.002 PMC4688223

[42] Jain JP , Chitkara D , Kumar N . Polyanhydrides as localized drug delivery carrier: an update. Expert Opin Drug Deliv. 2008;5(8):889‐907. 18712998 10.1517/17425247.5.8.889

[43] Jain JP , Modi S , Domb AJ , Kumar N . Role of polyanhydrides as localized drug carriers. J Control Release. 2005;103(3):541‐563. 15820403 10.1016/j.jconrel.2004.12.021

[44] Gopferich A , Tessmar J . Polyanhydride degradation and erosion. Adv Drug Deliv Rev. 2002;54(7):911‐931. 12384315 10.1016/s0169-409x(02)00051-0

[45] Sabharwal R , Weiss RM , Zimmerman K , Domenig O , Cicha MZ , Chapleau MW . Angiotensin‐dependent autonomic dysregulation precedes dilated cardiomyopathy in a mouse model of muscular dystrophy. Exp Physiol. 2015;100(7):776‐795. 25921929 10.1113/EP085066 PMC4505616

[46] Sabharwal R , Mason BN , Kuburas A , Abboud FM , Russo AF , Chapleau MW . Increased receptor activity‐modifying protein 1 in the nervous system is sufficient to protect against autonomic dysregulation and hypertension. J Cereb Blood Flow Metab. 2019;39(4):690‐703. 29297736 10.1177/0271678X17751352 PMC6446426

[47] Espinosa‐Cotton M , Rodman Iii SN , Ross KA , et al. Interleukin‐1 alpha increases anti‐tumor efficacy of cetuximab in head and neck squamous cell carcinoma. J Immunother Cancer. 2019;7(1):79. 30890189 10.1186/s40425-019-0550-z PMC6425573

[48] Chen L , Apte RN , Cohen S . Characterization of PLGA microspheres for the controlled delivery of IL‐1 alpha for tumor immunotherapy. J Control Release. 1997;43(2–3):261‐272.

[49] Katti DS , Lakshmi S , Langer R , Laurencin CT . Toxicity, biodegradation and elimination of polyanhydrides. Adv Drug Deliv Rev. 2002;54(7):933‐961. 12384316 10.1016/s0169-409x(02)00052-2

[50] Shen E , Kipper MJ , Dziadul B , Lim MK , Narasimhan B . Mechanistic relationships between polymer microstructure and drug release kinetics in bioerodible polyanhydrides. J Control Release. 2002;82(1):115‐125. 12106982 10.1016/s0168-3659(02)00125-6

[51] Waldmann TA . Cytokines in cancer immunotherapy. Cold Spring Harb Perspect Biol. 2018;10(12):a028472. 29101107 10.1101/cshperspect.a028472 PMC6280701

[52] Pires IS , Hammond PT , Irvine DJ . Engineering strategies for immunomodulatory cytokine therapies ‐ challenges and clinical Progress. Adv Ther (Weinh). 2021;4(8):2100035. 34734110 10.1002/adtp.202100035 PMC8562465

[53] Veltri S , Smith JW 2nd . Interleukin 1 trials in cancer patients: a review of the toxicity, antitumor and hematopoietic effects. Stem Cells. 1996;14(2):164‐176. 8991536 10.1002/stem.140164

[54] Li R , Mukherjee MB , Lin J . Coordinated regulation of myeloid‐derived suppressor cells by cytokines and chemokines. Cancers (Basel). 2022;14(5):1236. 35267547 10.3390/cancers14051236 PMC8909268

