# Supplementary figures and images for: Characterization of CPH:SA microparticle‐based delivery of interleukin‐1 alpha for cancer immunotherapy
Source: Bioeng Transl Med. 2022 Dec 7;8(3):e10465. doi: 10.1002/btm2.10465 (PMC10189482; doi:10.1002/btm2.10465)

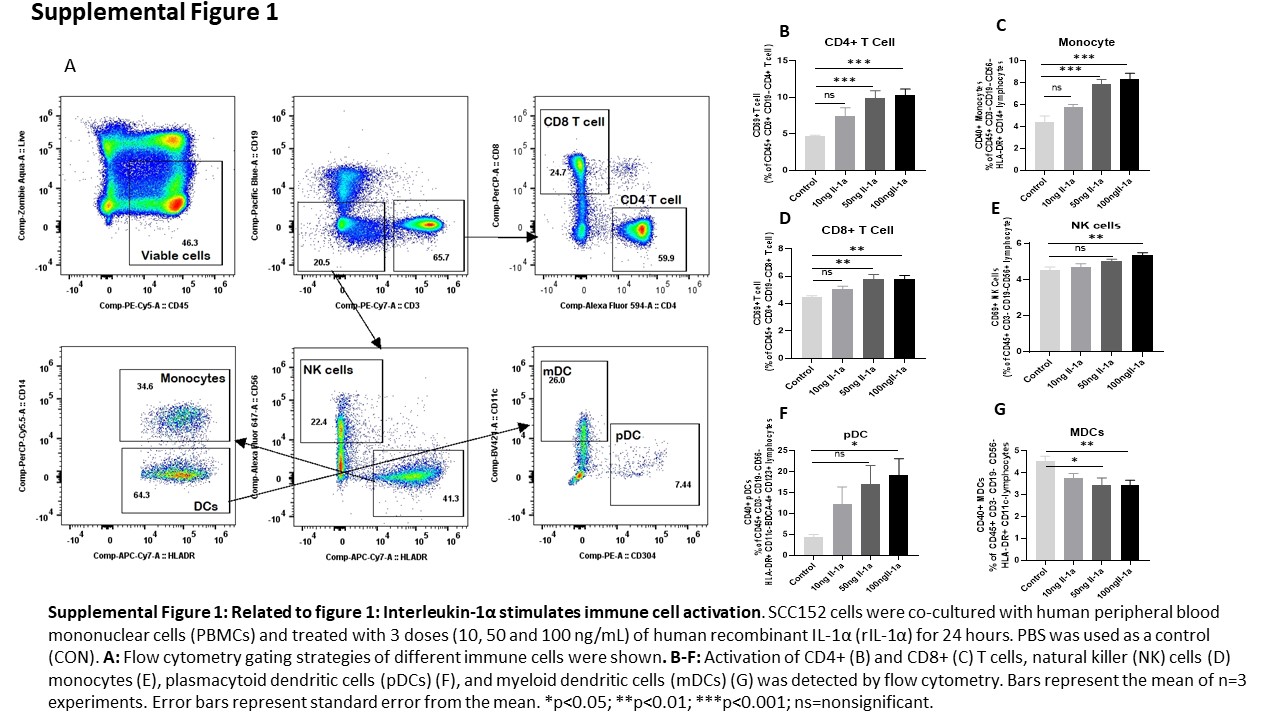

Supplement: Supplementary file 1 — Figure S1: Supporting information. [file BTM2-8-e10465-s001.jpg]

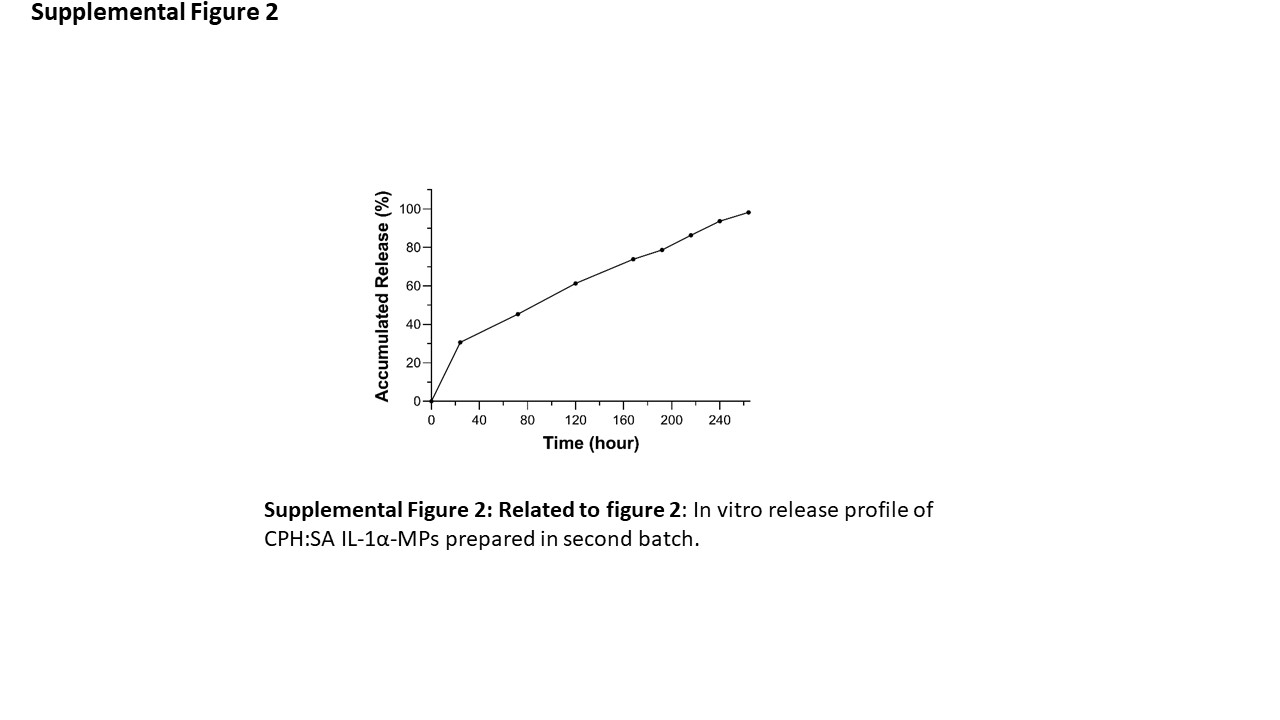

Supplement: Supplementary file 2 — Figure S2: Supporting information. [file BTM2-8-e10465-s004.jpg]

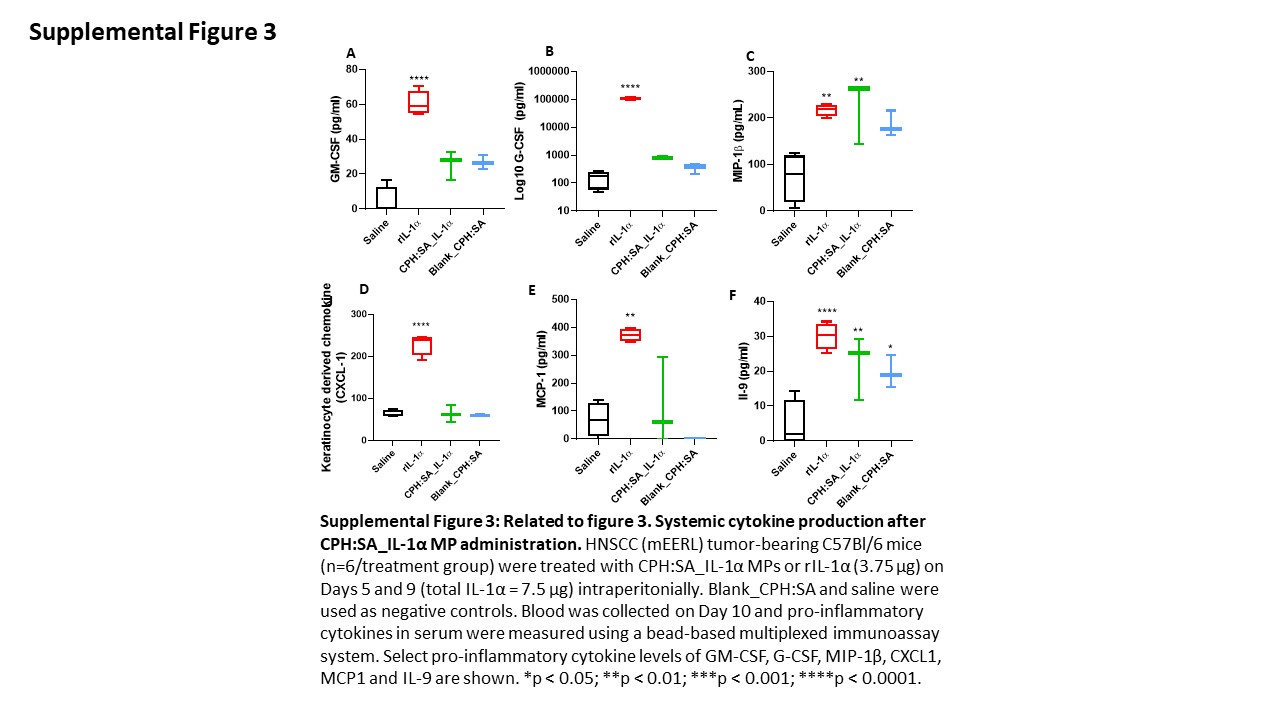

Supplement: Supplementary file 3 — Figure S3: Supporting information. [file BTM2-8-e10465-s003.jpg]
