# Supplementary material for: Characterization of CPH:SA microparticle‐based delivery of interleukin‐1 alpha for cancer immunotherapy
Source: Bioeng Transl Med. 2022 Dec 7;8(3):e10465. doi: 10.1002/btm2.10465 (PMC10189482; doi:10.1002/btm2.10465)
Supplement: Supplementary file 4 — Figure S4: Supporting information. [file BTM2-8-e10465-s002.pptx]

## Slide 1
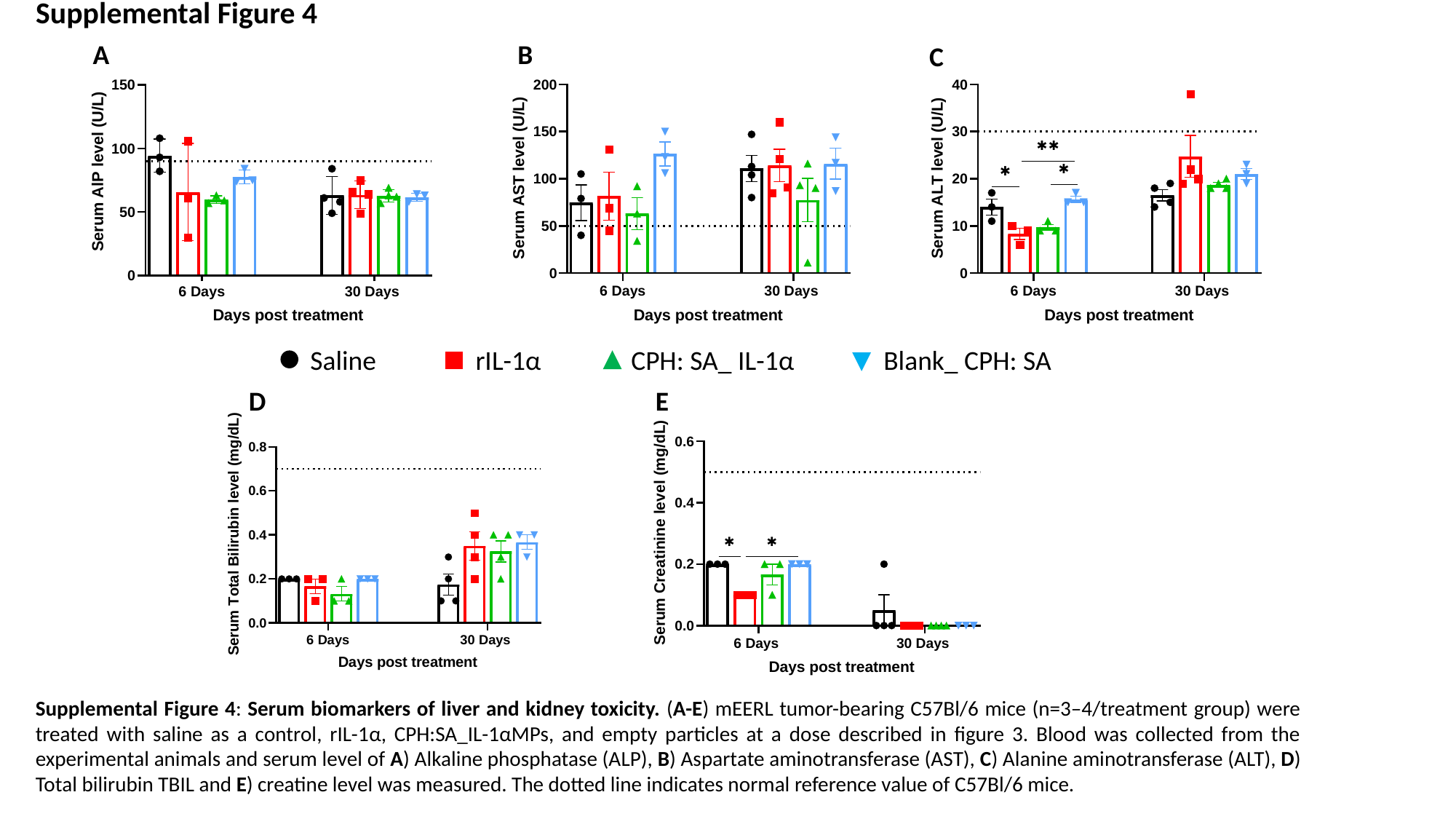

Supplemental Figure 4
A
B
C
Saline
rIL-1α
CPH: SA_ IL-1α
Blank_ CPH: SA
D
E
Supplemental Figure 4: Serum biomarkers of liver and kidney toxicity. (A-E) mEERL tumor-bearing C57Bl/6 mice (n=3–4/treatment group) were treated with saline as a control, rIL-1α, CPH:SA_IL-1αMPs, and empty particles at a dose described in figure 3. Blood was collected from the experimental animals and serum level of A) Alkaline phosphatase (ALP), B) Aspartate aminotransferase (AST), C) Alanine aminotransferase (ALT), D) Total bilirubin TBIL and E) creatine level was measured. The dotted line indicates normal reference value of C57Bl/6 mice.
